# BD MAX Enteric Bacterial, Bacterial Plus, and Virus Panels for Diagnosis of Acute Infectious Gastroenteritis: a Cost-Benefit Analysis

**DOI:** 10.1128/spectrum.00880-22

**Published:** 2022-09-07

**Authors:** Josep Ferrer, Estela Giménez, Diego Carretero, Javier Buesa, Francisco Morillas, Rafael Granell, Amadeo Fuenmayor, David Navarro, Eliseo Albert

**Affiliations:** a Microbiology Service, Clinic University Hospital, INCLIVA Health Research Institute, Valencia, Spain; b Department of Applied Economics, School of Economics, University of Valencia, Valencia, Spain; c Department of Microbiology, School of Medicine, University of Valencia, Valencia, Spain; Mycology Laboratory, Wadsworth Center

**Keywords:** cost-benefit analysis, acute gastroenteritis diagnosis, syndromic PCR panel, Markov model, BD MAX

## Abstract

Economic assessment is required to gauge the value of implementing PCR syndromic platforms in the microbiology laboratory for the diagnosis of community-acquired acute gastroenteritis (AGE) in pediatric and adult in- and outpatients. A cost-benefit analysis was conducted from a health care system perspective using BD MAX Enteric Bacterial, Bacterial Plus, and Virus panels. Two 6-month periods were selected, in which either conventional procedures (in 2017) or BD MAX PCR multiplex panels (in 2018) were used. We retrospectively reviewed medical records of all patients with positive results and a representative sample of negative ones. A Markov model was used to represent transition probabilities between different health care states from time of stool microbiological study until completion of AGE-episode-associated health care. A total of 1,336 medical records were reviewed (829 in 2018 and 507 in 2017), showing overall a significantly higher positivity rate in 2018 than in 2017 (26% versus 6%, *P < *0.001). The total cost per individual associated with health care for AGE was €314 in 2018 and €341 in 2017; when we only considered the pediatric cohort, the figures were €271 and €456, respectively. Using Tornado sensitivity analyses, we found that the three variables that most influenced the model in descending order of weight were the probability of longer hospital stays, the probability of returning to the emergency room (ER), and the probability of hospitalization from the ER. Use of BD MAX enteric PCR platforms for the diagnosis of community-acquired AGE instead of a non-PCR-based conventional approach results in an incremental benefit from a health care perspective in the general population, particularly children.

**IMPORTANCE** The implementation of multiplex molecular panels allows microbiological laboratories to quickly, sensitively, and accurately diagnose acute infectious gastroenteritis. This methodology therefore allows faster decisions regarding treatment and infection control measures. Economic evaluations are required to gauge the value of implementing these syndromic PCR platforms in a community-based acute gastroenteritis setting. We studied the potential clinical and cost benefits, in terms of both their impact on laboratory costs and the subsequent costs of managing patients.

## INTRODUCTION

Acute infectious gastroenteritis (AGE) is a major cause of childhood morbidity, frequently requiring medical consultation and hospitalization (1), which results in a high financial and health care burden (1, 2). The estimated incidence of AGE in Europe is more than 0.5 to 2 episodes/year in children under 3 years of age (3). The Infectious Diseases Society of America recommends performing stool cultures only in cases of invasive diarrhea or severe illness, as defined by the presence of one or more of the following: fever, dehydration, systemic symptoms, severe abdominal pain, or bloody or mucoid stools (4). The use of PCR multiplex panels has increased in popularity in recent years for AGE diagnosis; while molecular assays increase the diagnostic yield compared to culture methods, it remains unclear whether their use has a major impact on patient management and health care system-associated costs (5). Previous studies have shown reductions in time to empirical antibiotic therapy initiation, length of hospital stay, and isolation and improvement in the prescription of targeted antibiotic therapy when using multiplex PCR panels for the diagnosis of nosocomial or combined community- and hospital-acquired AGE (6–10). However, recent systematic reviews and a meta-analysis addressing this issue were not conclusive regarding the cost-effectiveness of this diagnostic strategy for either community-acquired or nosocomial AGE (11, 12). To shed light on this issue, we evaluated the cost-benefit ratio, from a health care system perspective, of implementing BD MAX enteric PCR panels (BD Diagnostics, Sparks, MD) for diagnosis of community-acquired AGE in either hospitalized or nonhospitalized children and adults.

## RESULTS

A total of 1,336 medical records were reviewed: (i) 829 from 2018, 522 (62.9%) of which corresponding to all positive samples of the selected semester in 2018 and 307 (37%) of which resulting from sampling among 1,483 negative samples, and (ii) 507 from 2017, 162 (32%) of which corresponding to all positive samples of the same semester in 2017 and 345 (68%) of which resulting from sampling among 2,388 negative samples. The distribution of microbiological study results across the different groups and clusters within the two study periods is shown in Table 1. A significantly higher positivity rate was observed in 2018 than in 2017 (26% versus 6%, *P < *0.001) (Table 2), which was not attributable to seasonal differences between the two sampling periods. As expected, the diagnostic yield was higher in 2018 than in 2017 for most bacteria and viruses screened. This was particularly striking for Campylobacter spp. and, to a lesser extent, for *Shigella* spp. and *Vibrio* spp. The opposite was observed for Salmonella and rotavirus AGE in children. The number of coinfections was significantly greater in 2018 than in 2017 (14% versus 6%, *P* < 0.001). The frequency and distribution for each causative agent of AGE according to patient’s age and study period are shown in Table 3.

**TABLE 1:** Microbiological study results across different patient groups and clusters within two study periods

Patient group	No. (%) in 2017			No. (%) in 2018		
	Total	Positive	Negative	Total	Positive	Negative
Outpatients						
Total	205 (100)	57 (27.8)	148 (72.2)	351 (100)	239 (68.1)	112 (31.9)
Pediatric	108 (52.7)	49 (23.9)	59 (28.8)	224 (63.8)	190 (54.1)	34 (9.7)
Immunocompetent	108 (52.7)	49 (23.9)	59 (28.8)	223 (63.5)	189 (53.8)	34 (9.7)
Immunosuppressed	0 (0)	0 (0)	0 (0)	1 (0.3)	1 (0.3)	0 (0)
Adults (<65 yrs)	65 (31.7)	6 (2.9)	59 (28.8)	87 (24.8)	39 (11.1)	48 (13.7)
Immunocompetent	64 (31.2)	6 (2.9)	58 (28.3)	86 (24.5)	38 (10.8)	48 (13.7)
Immunosuppressed	1 (0.5)	0 (0)	1 (0.5)	1 (0.3)	1 (0.3)	0 (0)
Elderly (>65 yrs)	32 (15.6)	2 (1.0)	30 (14.6)	40 (11.4)	10 (2.8)	30 (8.5)
Immunocompetent	31 (15.1)	2 (1.0)	29 (14.1)	39 (11.1)	10 (2.8)	29 (8.3)
Immunosuppressed	1 (0.5)	0 (0)	1 (0.5)	1 (0.3)	0 (0)	1 (0.3)
Hospitalized patients						
Total	302 (100)	105 (34.8)	197 (65.2)	478 (100)	283 (59.2)	195 (40.8)
Pediatric	118 (39.1)	68 (22.5)	50 (16.6)	150 (31.4)	115 (24.1)	35 (7.3)
Immunocompetent	76 (25.2)	64 (21.2)	12 (4.0)	114 (23.8)	107 (22.4)	7 (1.5)
Immunosuppressed	42 (13.9)	4 (1.3)	38 (12.6)	36 (7.5)	8 (1.7)	28 (5.9)
Adults (<65 yrs)	112 (37.1)	27 (8.9)	85 (28.1)	189 (39.5)	106 (22.2)	83 (17.4)
Immunocompetent	59 (19.5)	23 (7.6)	36 (11.9)	113 (23.6)	80 (16.7)	33 (6.9)
Immunosuppressed	53 (17.5)	4 (1.3)	49 (16.2)	76 (15.9)	26 (5.4)	50 (10.5)
Elderly (>65 yrs)	72 (23.8)	10 (3.3)	62 (20.5)	139 (29.1)	62 (13.0)	77 (16.1)
Immunocompetent	33 (10.9)	8 (2.6)	25 (8.3)	75 (15.7)	48 (10.0)	27 (5.6)
Immunosuppressed	39 (12.9)	2 (0.7)	37 (12.3)	64 (13.4)	14 (2.9)	50 (10.5)

**TABLE 2:** Number of tests performed and frequency of causative agents of AGE within two study periods

Parameter	No. (%) in:		*P*
	2017	2018	
Total studies performed in the study period	2,550	2,005	
Positive results	162 (6.4)	522 (26.0)	<0.001
Negative results	2,388 (93.6)	1,483 (74.0)	<0.001
Studies included by sampling method	507	829	
Bacteria	308 (60.7)	354 (42.7)	<0.001
Virus	8 (1.6)	18 (2.2)	0.577
Bacteria and virus	191 (37.7)	457 (55.1)	<0.001
Bacteria	108 (66.7)	310 (59.4)	0.117
*Campylobacter* spp.	15 (13.9)	165 (53.2)	<0.001
Enterotoxigenic *E. coli*	7 (6.5)	27 (8.7)	0.599
*Plesiomonas shigelloides*	0	4 (1.3)	0.236
*Salmonella* spp.	73 (67.6)	86 (27.7)	<0.001
Shiga toxin genes		14 (4.5)	
*Shigella* spp.	0	14 (4.5)	0.025
*Vibrio* spp.	0	14 (4.5)	0.025
*Yersinia enterocolitica*	3 (2.8)	7 (2.3)	0.951
Other	9 (8.3)	0	<0.001
Virus	57 (35.2)	229 (43.9)	0.062
Adenovirus	30 (52.6)	34 (14.8)	<0.001
Astrovirus		45 (19.7)	
Norovirus		95 (41.5)	
Rotavirus	32 (56.1)	10 (4.4)	<0.001
Sapovirus		90 (39.3)	
Coinfections	9 (5.6)	73 (14.0)	0.006
Bacteria	0	18 (24.7)	0.092
Virus	6 (66.7)	38 (52.0)	0.635
Bacteria and virus	3 (33.3)	17 (23.3)	0.802
			
Recovery of bacteria by conventional culture		168 (50.8)^*a*^	

a Number of strains: Campylobacter spp., 68 (41.2%); Enterotoxigenic E. coli, 21 (77.8%); Salmonella spp., 63 (73.3%); *Shigella* spp., 9 (64.3%); *Vibrio* spp., 5 (35.7%); Yersinia enterocolitica, 2 (28.6%).

**TABLE 3:** Frequency and distribution of each causative agent of AGE according to patient age within two study periods

Infection type	No. (%) of patients in 2017			No. (%) of patients in 2018			*P*
	Pediatric	Adults	Elderly	Pediatric	Adults	Elderly	
Positive tests for each study period	117 (72.2)	33 (20.4)	12 (7.4)	305 (58.4)	145 (27.8)	72 (13.8)	0.075
Microbiological results							
Virus							
Norovirus				69 (72.6)	15 (15.8)	11 (11.6)	
Sapovirus				83 (92.2)	6 (6.7)	1 (1.1)	
Astrovirus				37 (82.2)	5 (11.1)	3 (6.7)	
Adenovirus	26 (86.7)	4 (13.3)	0 (0)	25 (73.6)	6 (17.6)	3 (8.8)	0.004
Rotavirus	26 (86.7)	6 (13.3)	0 (0)	8 (80.0)	0 (0)	2 (20.0)	<0.001
Bacteria							
*Salmonella* spp.	46 (63.0)	17 (23.3)	10 (13.7)	45 (52.3)	26 (30.2)	15 (17.4)	0.387
*Campylobacter* spp.	9 (60.0)	5 (33.3)	1 (6.7)	81 (49.1)	53 (32.1)	31 (18.8)	0.036
ETEC Shiga toxin genes *and Shigella* spp.	7 (100.0)	0 (0)	0 (0)	11 (20.0)	38 (69.1)	6 (10.9)	<0.001
*Plesiomonas*, *Vibrio*, and *Yersinia* spp.	7 (53.8)	5 (38.5)	1 (7.7)	6 (24.0)	14 (56.0)	5 (20.0)	<0.001
Coinfections	5 (62.5)	3 (37.5)	0 (0)	53 (72.6)	15 (20.6)	5 (6.8)	0.002

Regarding stool sample origin, in 2017, 92 microbiological studies were requested from primary care, 169 were from specialized care, 146 were from the emergency room (ER), and 100 were from the hospital, compared to 125, 311, 265, and 128, respectively, in 2018. The numbers of patients who returned to primary or specialized care for test results and who did not need any further health care assistance were 86 (17%) and 133 (26%), respectively, in 2017, and 113 (14%) and 244 (29%), respectively, in 2018 (*P > *0.50). In 2017, patients requested care from the ER a total of 188 times; of these, 27 patients returned to the ER once more, 9 patients returned twice, 3 patients returned three times, and 1 patient returned five times. In 2018, there were a total of 343 episodes requested from emergency care; only 11 patients returned once. The proportion of revisits to the ER was significantly higher in 2017 than in 2018 (*n* = 40 [21.8%] versus *n* = 11 [3%], *P* < 0.001).

There were 135 and 179 hospital admissions in 2017 and 2018, respectively; of these, 48 and 51, respectively, were directly related to the diarrhea episode. The median length of hospital stay (considering only related episodes) was 120 h (interquartile range, 72 to 216 h) in 2017, and 72 h (interquartile range, 72 to 168 h) in 2018.

### Markov model: differential transition probabilities.

All possible transitions between Markov states until discharge and their probabilities for each state by period are reported in Table 4. We incorporated initial test price values of €6.5 and €32, applicable to the diagnostic procedures in 2017 and 2018, respectively. The health care costs per patient in the general population calculated by the Markov model and associated with the transition between health care states for patients with AGE were €314 in 2018 and €341 in 2017. When considering the pediatric population separately, the total costs per patient were €271 and €456 for 2018 and 2017, respectively. Implementing the new molecular method afforded a benefit of €27 when all patients were analyzed and a benefit of €185 when only the pediatric population was included in the analysis.

**TABLE 4:** Possible transitions between Markov states and their probabilities

Parameter	All (%)		Pediatric population (%)	
	2017	2018	2017	2018
Initial probability				
Emergency room	28.8	31.97	31.14	27.27
Hospital	19.72	15.44	12.72	3.74
Primary care	18.15	15.08		
Specialized care	33.33	37.52	56.14	68.99
Transition probabilities				
Emergency room				
Emergency room	17.90	3.11	24.06	3.12
Hospitalization	15.28	14.12	18.05	10.00
Primary care	26.65	35.88		
Specialized care	34.93	42.37	57.89	86.88
Discharge	5.24	4.52	0	0
Hospital				
Emergency room	0	0	0	0
Hospitalization	64.00	55.69	68.45	74.14
Primary care	0	0		
Specialized care	0	0	0	0
Discharge	36.00	44.31	31.55	25.86
Primary care				
Emergency room	2.52	3.29		
Hospitalization	0	0		
Primary care	35.98	30.96		
Specialized care	0	0		
Discharge	61.50	65.75		
Specialized care				
Emergency room	9.42	9.35	14.64	8.80
Hospitalization	0	0.14	0	0
Primary care	0	0		
Specialized care	34.82	34.70	47.80	34.05
Discharge	55.76	55.81	37.56	57.15

The median costs and 90% confidence intervals determined by the Monte Carlo sampling method for 2017 and 2018 were €340 (€321 to €358) and €315 (€302 to €327), respectively, for the general population and €456 (€435 to €474) in 2017 and €269 (€255 to €285) in 2018 for the pediatric population alone. The distribution of expected values for each sample according to the Monte Carlo simulation can be seen in Fig. S5 in the supplemental material.

### Sensitivity analysis.

We performed Tornado sensitivity analysis to evaluate the relative weight of the variables included in the model, as well as to find the critical value of the main variables that changed the direction of the analysis. As shown in Fig. S6, the three variables that most influenced the model, in descending order of weight, were the probability of extending the hospital stay by another day, the probability of an ER revisit, and the probability of hospitalization from the ER.

## DISCUSSION

Use of multiplex PCR panels for the diagnosis of a number of infectious syndromes, including meningitis/encephalitis, bacteremia, and respiratory infections, may result in overall savings due to a lower demand for health care services and a more judicious use of antibiotics (13–19). Evidence supporting this has also been gathered by several studies for nosocomial AGE, including AGE caused by Clostridioides difficile or combined hospital- and community-acquired AGE. For example, in an 8-month parallel diagnostic study aimed at measuring the potential economic benefits of testing hospitalized patients with the Luminex xTAG Gastrointestinal Pathogen Panel (GPP) compared to conventional diagnostic approaches, Goldenberg et al. found that use of GPP resulted in savings of £66,765 attributable to reduced isolation times, which clearly offset the additional laboratory testing costs (6). In turn, Beal et al. (7) found that using the BioFire FilmArray gastrointestinal panel for the diagnosis of AGE episodes seemingly including both community- and hospital-acquired episodes reduced overall health care costs by $293.61 per patient tested by decreasing the number of days on antibiotic(s) per patient, the number of imaging studies, and the average time from stool culture collection to discharge. Likewise, implementing the BioFire FilmArray gastrointestinal panel, also in apparently mixed cohorts, was associated with reduced use of endoscopy, abdominal radiology and antibiotic prescribing in two other studies (9, 10), although the precise extent of the saving was not provided. Nonetheless, as previously indicated (11, 12, 20), there are limited data available on the cost-effectiveness of implementing multiplex GI panels for the diagnosis of community-acquired AGE, thus excluding those due to Clostridioides difficile.

Herein, we carried out a cost-benefit analysis, in terms of health care, of implementing BD MAX enteric panels for diagnosing community-acquired AGE in a cohort comprising both children and adults, attended at either primary care centers or hospitals. We chose a Markov modeling approach, commonly used in cost-benefit and cost-effectiveness analyses, due to its simplicity and out-of-sample forecasting accuracy. Note that the Spanish public system provides free basic health care to those who contribute to the Spanish social security system and their families, so expenses incurred by the patients are paid collectively by society as a whole. The following factors are also relevant to data interpretation. First, we assumed similar indirect costs associated with diagnostic processes across both study periods. Second, since only loose or watery stool specimens were processed in both study periods, we interpreted any enteropathogen isolation/detection as a true positive (causative of the AGE episode).

In agreement with previous reports (20–23), our data indicated that the use of BD MAX panels increased the overall diagnostic yield compared to non-PCR-based methods, which was particularly noticeable for most enteropathogenic bacteria (especially Campylobacter spp.); however, we were not able to demonstrate whether the differences found in the bacteria recovered between the 2 years is attributable to the different sensitivity between the diagnostic methods or specific outbreaks in each case. Despite this, Salmonella spp. and rotavirus were identified as the cause of AGE in children less frequently in 2018 than 2017. This unexpected result was plausibly related to differences in incidence of AGE outbreaks due to these agents across the two study periods, but this assumption could not be confirmed. Despite significant additional direct costs, the use of BD MAX enteric PCR panels afforded a benefit of €27 per AGE episode when all patients were collectively considered for the analyses and a benefit of €185 when only the pediatric population was included. According to our sensitivity analysis, overall savings were mostly associated with reductions in hospital stay and number of ER visits and, to a lesser extent, the probability of hospitalization from the ER.

Our cost-benefit study focused on events occurring between the request for microbiological analysis and end of patient follow-up, which in most cases coincided with receipt of results. In this context, we speculate that the difference in terms of net gain across the two study periods derives from patient uncertainty while waiting for results, which likely prompted them to seek further health care assistance. Insurance companies have tightened reimbursement for syndromic panels because they consider medical intervention to be largely unnecessary in AGE due to viral or bacterial etiologies. However, we focused on reducing the uncertainty between analysis request and obtaining the results. This situation could decrease the health care demand regardless of AGE severity and lead to a reduction in cost. Likewise, the most likely explanation for shorter patient hospitalization in 2018 than in 2017 is that knowing the etiology of AGE avoided further medical explorations and prompted early release. Nevertheless, the study was not designed to gauge the potential impact of either increased diagnostic yield and faster turnaround times with the BD MAX enteric PCR platforms, nor of the enteropathogens detected, on the overall management of AGE and associated costs (antibiotic therapy, complementary imaging studies, etc.), which appear to be substantial according to previous studies (6, 7, 9, 10).

Our study should be interpreted in light of certain limitations. First, a degree of heterogeneity and uncertainty is inherent to the use of Markov models, which extrapolate real-life experiences to a model that incorporates a series of concrete states defined by a limited number of variables. Second, we used average costs for each Markov state, instead of a more precise micro-cost analysis approach. Third, the exact time dedicated to technical procedures in 1 year, and the other was not calculated, but we considered it comparable. The specific micro-cost associated with microbiological laboratory procedures, such as the cost of a diffusion disk test, was also not considered. This limitation must be taken into account to extrapolate the results to other environments. Fourth, regarding the sampling method, all positive tests were sampled in each study period, while a representative number of negative samples was gathered in the different clusters, with an overestimated number in underrepresented cohorts. As a result, the number of samples included in the analyses was higher in 2018 than in 2017. Nevertheless, we assume that the number of AGE episodes was not substantially dissimilar across the study periods, implying that the difference in diagnostic yield reflected the greater sensitivity of the molecular device. In conclusion, the use of BD MAX enteric PCR platforms instead of conventional nonmolecular approaches for the diagnosis of community-acquired AGE results in an incremental benefit in the general population, especially in children.

## MATERIALS AND METHODS

### Study population.

We retrospectively reviewed medical records from in- and outpatients attended at the Health Department Clinico-Malvarrosa of Valencia (which includes 16 health care centers and two hospitals attending a population of 341,662) presenting with community-acquired AGE, in whom etiological diagnosis was sought within two 6-month periods: July to December 2017, during which standard stool cultures and virus antigen-based detection assays were used for AGE diagnosis, and July to December 2018, during which BD MAX enteric PCR panels were used for this purpose. There was no transition period during which both strategies were used in parallel. Requesters were not given the opportunity to choose the diagnostic strategy to be pursued. Laboratory-based algorithms were followed within both study periods. Both diagnostic approaches were in place from Monday to Saturday. We excluded all (hospitalized or nonhospitalized) patients with nosocomial AGE (manifesting at least 72 h after hospitalization) or clinical suspicion of Clostridioides difficile infection. While all patients returning positive results were considered for the analyses, a sampling method was applied to select patients with negative results, based on the real sample size within the study periods, assuming an error rate of 1 to 2% as described below.

### Ethical research.

This study was performed in line with the principles of the Declaration of Helsinki and approved by the Research Ethics Committee of the Clinic University Hospital of Valencia INCLIVA, Spain (2020/008), who exempted us from obtaining informed consent because of the study’s retrospective nature. All data were anonymized before analysis.

### Sampling method.

A total of 24 clusters were generated for each study period using a stratified sampling method, which combined the following clinical and demographic variables of the study population: pediatric versus adult versus elderly, hospital versus primary health care, immunosuppressed versus immunocompetent, and positive versus negative microbiological result. To avoid potential inaccuracies due to underrepresented clusters, the sample size in each cluster was estimated by the probability proportional to size (PPS) approach (30 to 70%) as previously recommended (12, 24, 25), in which 30% of the sample size in each cluster was common to all clusters, and 70% proportional to the size of each cluster in the original population. In each cluster, patients were randomly selected in both periods (2017 and 2018) by assigning random numbers using R software (R-Project; R Foundation for Statistical Computing, Vienna, Austria [http://www.R-project.org]).

All data (laboratory testing, days of hospitalization, emergency department visits, medical consultations, etc.) were reviewed from the first requested microbiological study until the AGE episode was resolved (end of follow-up).

### Laboratory procedures. (i) Diagnostic approach to AGE from July to December 2017.

Loose, watery, or unformed stool samples (type 5 to 7 stools according to the Bristol Stool Chart) were processed according to a laboratory protocol in place, following Spanish Society of Microbiology guidelines (26), as summarized in Fig. S1 in the supplemental material. Fresh samples were initially examined for the presence of red and white blood cells (polymorphonuclear and mononuclear cells) by light microscopy in order to provide the requester with information about potential involvement of invasive enteropathogens. Specimens were subsequently seeded in different selective or differential culture media, including Campylobacter agar, Salmonella-*Shigella* agar, MacConkey agar, blood agar, *Yersinia* selective agar and selenite enrichment broth (subcultured for 24 h in Salmonella-*Shigella* agar), all purchased from Becton Dickinson (Rutherford, NJ) to investigate the presence of Campylobacter spp., Salmonella spp., Escherichia coli, *Aeromonas* spp., *Plesiomonas* spp., *Yersinia* spp., *Vibrio* spp., and *Shigella* spp. Bacterial species were identified by matrix-assisted laser desorption/ionization-time of flight mass spectrometry (MALDI-TOF-MS) (Bruker Daltonics, Billerica, MA). Turnaround times ranged between 24 and 48 h for positive results, whereas they usually took 48 h for negative results. Upon isolating and identifying any of the aforementioned bacteria, antimicrobial susceptibility testing was performed. In addition, stool samples from patients under 5 years of age and elderly patients (>80 years) were systematically tested by lateral-flow immunochromatography (LFIC; CerTest Biotec, Zaragoza, Spain) for the presence of rotavirus and adenovirus antigens. LFIC was occasionally performed in patients of any age upon medical prescription (i.e., in immunosuppressed patients).

### (ii) Diagnostic approach to AGE from July to December 2018.

The BD MAX enteric bacterial panel kit (BD Life Sciences, Sparks, MD) run on a BD MAX platform can detect Salmonella spp., *Shigella* spp., Campylobacter jejuni*/coli*, and Shiga toxin genes, while the BD MAX extended enteric bacterial panel (which comes as an additional master mix to be used in conjunction with the BD MAX enteric bacterial panel) detects Yersinia enterocolitica, enterotoxigenic E. coli (ETEC), *Vibrio* spp. (V. vulnificus, V. parahaemolyticus, and V. cholerae), and Plesiomonas shigelloides. The BD MAX enteric viral panel enables detection of rotavirus, adenovirus, sapovirus, norovirus, and astrovirus. BD MAX panels were used according to the manufacturer’s instructions. As shown in Fig. S2, an age-based criterion similar to that in use during the year before implementing the BD MAX panels was used for testing. Stools from all patients were systematically tested for the presence of bacteria using both panels in conjunction. Note that no patients returning negative results via BD MAX enteric bacteria panels were retested (add-on) using the BD MAX enteric viral panel. Turnaround times ranged between 6 and 24 h for positive and negative results. Specimens testing positive for bacterial species were plated in the appropriate culture media. Recovered bacteria were identified by MALDI-TOF-MS and tested for antimicrobial susceptibility when appropriate.

### Model description.

We used a homogeneous Markov model to represent transition probabilities between different health care states. The model included five health care states for adult patients and four for pediatric ones (see Fig. S3 and S4 in the supplemental material, respectively), comprising all possible scenarios in which stool microbiological studies were ordered until resolution of the AGE episode. Only a single microbiological study per each complete Markov transition was considered. Any second study ordered for a given patient represented a new entry in the Markov model and passed through the different states until leaving the model, and there was a minimum 7-day interval between microbiological study requests in each patient. Five states were considered for adults: hospitalization, visit to the ER, visit to a specialized health care center or primary health care center, and end of follow-up (EOF). Four states were considered for children since these were always attended by specialized care doctors (pediatricians) at specialized healthcare centers. The initial probability of each state corresponded to the percentage of studies requested from each origin, except that of the EOF state. All states were reversibly connected to each other except for the EOF state, which was set as a nonreturn state. However, several states in this study were not connected in practice (for example, the probability of going to the ER from the hospital in 2017 was considered 0; see Table 3). The duration of each cycle was 24 h, and the total number of cycles was seven (maximum time until result). The transition probabilities between health care states were calculated according to the proportions of transitions observed in the study cohort and are assumed homogeneous in all cycles and also constant in each year.

### Variability study.

In order to estimate health care-associated costs in 1 year, the variability of the process in another and to evaluate the variance and robustness of the model, we made an approximation using the Monte Carlo simulation with 100 hypothetical samples and 1,000 repetitions.

### Costs and benefits.

For cost-benefit analysis, we used a health care perspective, that is, we focused on the costs and benefits incurred by the health care system. Health care costs per day associated with each Markov state, which included all direct and indirect estimated costs, were obtained from the Valencian Community Government database. Direct costs of the reagents for each diagnostic procedure, which included purchase charges, equipment maintenance, and proficiency testing, were obtained from the finance department of our hospital and applied to the first Markov cycle only, as shown in Table 5. Indirect costs associated with performing microbiological procedures were not taken into consideration, since, according to our estimates, the time spent by the laboratory technician to run 24 samples in the BD MAX system (and process samples testing positive for bacteria by conventional culture) was roughly equal to the total time spent on microscopic examination of the specimen, seeding in culture media, bacterial identification, antimicrobial susceptibility testing, and virus antigen testing for an equivalent number of specimens.

**TABLE 5:** Cost of patient care and reagents for the microbiological diagnosis of community-acquired AGE

Item	Cost (€)
Cost incurred per bacterial culture	
MacConkey agar	0.29
Salmonella shigella agar	0.19
Yersinia selective agar	0.28
Campylobacter agar	0.26
Blood agar	0.27
Selenite enrichment broth	0.52
MALDI-TOF-MS identification	1.2
Total	3.0
Cost incurred per antimicrobial susceptibility testing	
Kirby-Bauer method (disk diffusion test)	0.8
Costs incurred per virus detection	
Immunochromatography for rotavirus and adenovirus	3.5
Costs incurred per molecular biology diagnosis	
BD MAX enteric bacterial panel	11.0
BD MAX extended enteric bacterial panel	5.0
BD MAX enteric viral panel	16.0
Costs incurred per day	
Emergency room	189.0
Hospital	310.0
Primary care	29.0
Specialized care	50.0

The net gains (PROFIT) in each study period were calculated as follows: PROFIT2017 = BENEFITS2017 – COSTS2017; PROFIT2018 = BENEFITS2018 – COSTS2018. Assuming equal benefits for the two study periods, the differential impact can be calculated by NET PROFIT = COSTS2017 – COSTS2018.

### Software.

All analyses were performed using TreeAge Pro software package (version 2011, R1.0; TreeAge Software, Inc., Williamstown, MA).
