# Supplementary material for: BD MAX Enteric Bacterial, Bacterial Plus, and Virus Panels for Diagnosis of Acute Infectious Gastroenteritis: a Cost-Benefit Analysis
Source: Microbiol Spectr. 2022 Sep 7;10(5):e00880-22. doi: 10.1128/spectrum.00880-22 (PMC9603360; doi:10.1128/spectrum.00880-22)
Supplement: Supplemental file 1 — Fig. S1 to S6. Download spectrum.00880-22-s0001.pdf, PDF file, 0.4 MB [file spectrum.00880-22-s0001.pdf]

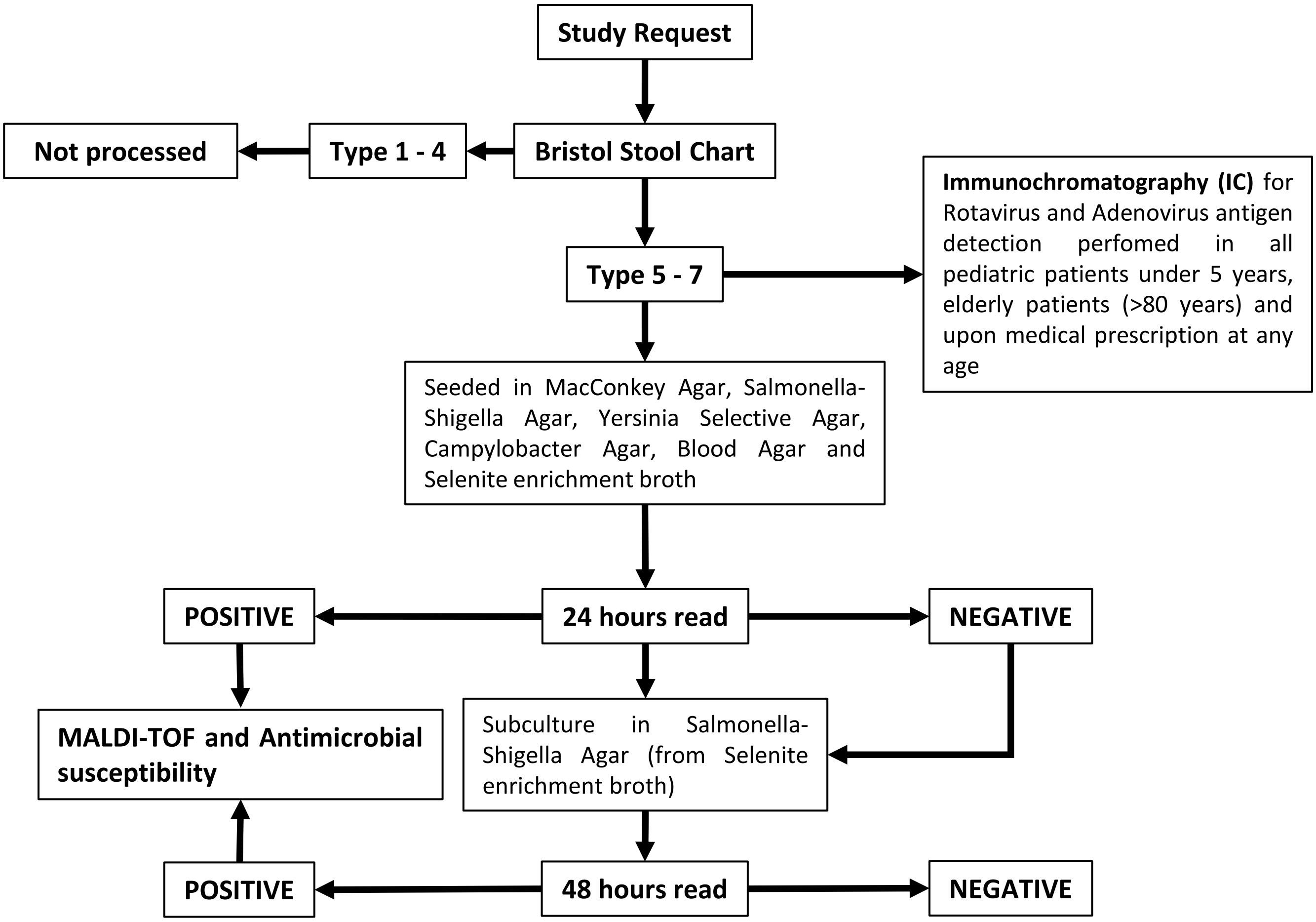

Supplementary Figure 1. Diagnostic algorithm during 2017 in which both culture-dependent and lateral flow device immunochromatography methods were used for the diagnosis bacterial and viral acute gastroenteritis.

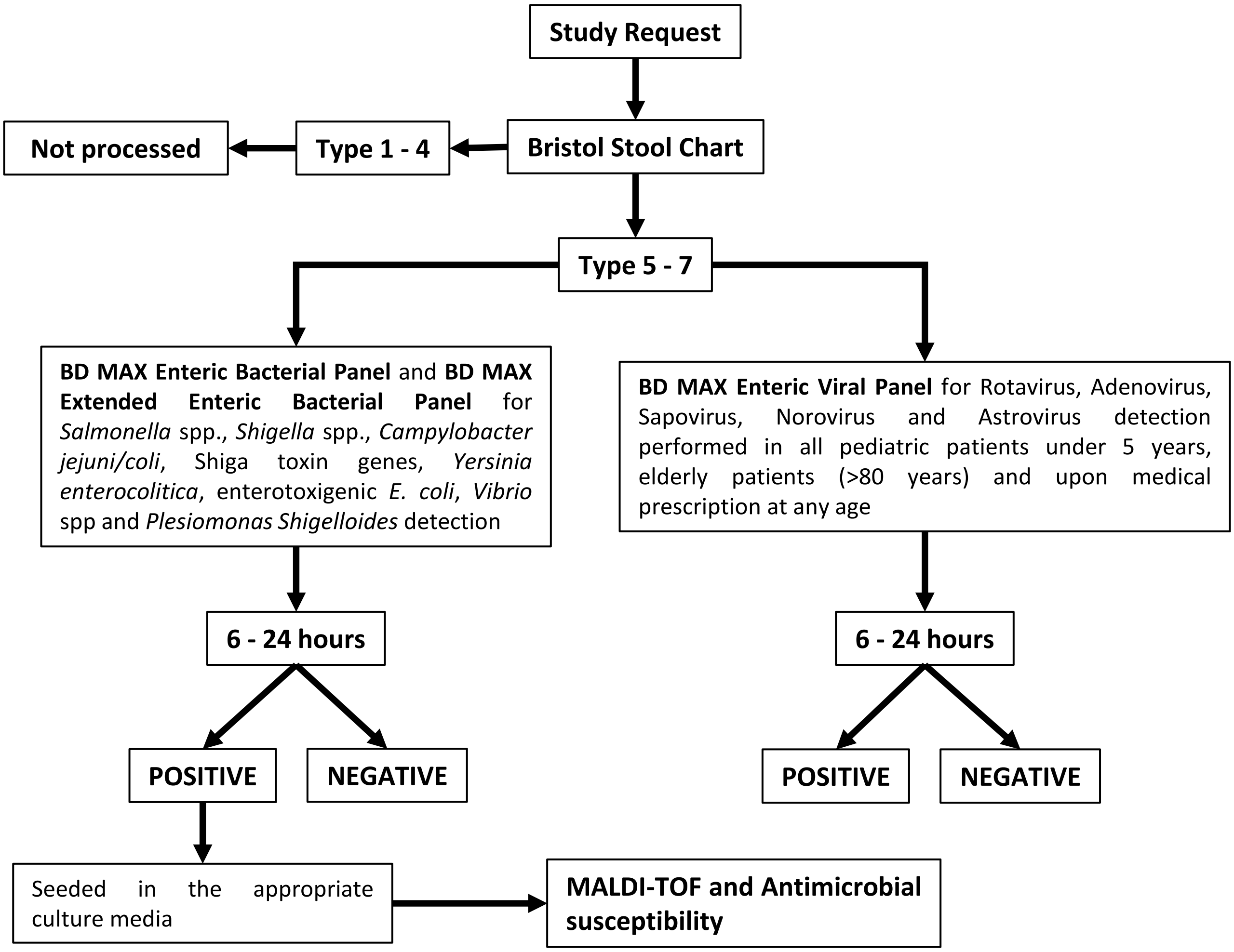

Supplementary Figure 2. Diagnostic algorithm during 2018 in which the BD Max TM PCR enteric panels were used for the diagnosis of baterial and viral acute gastroenteritis.

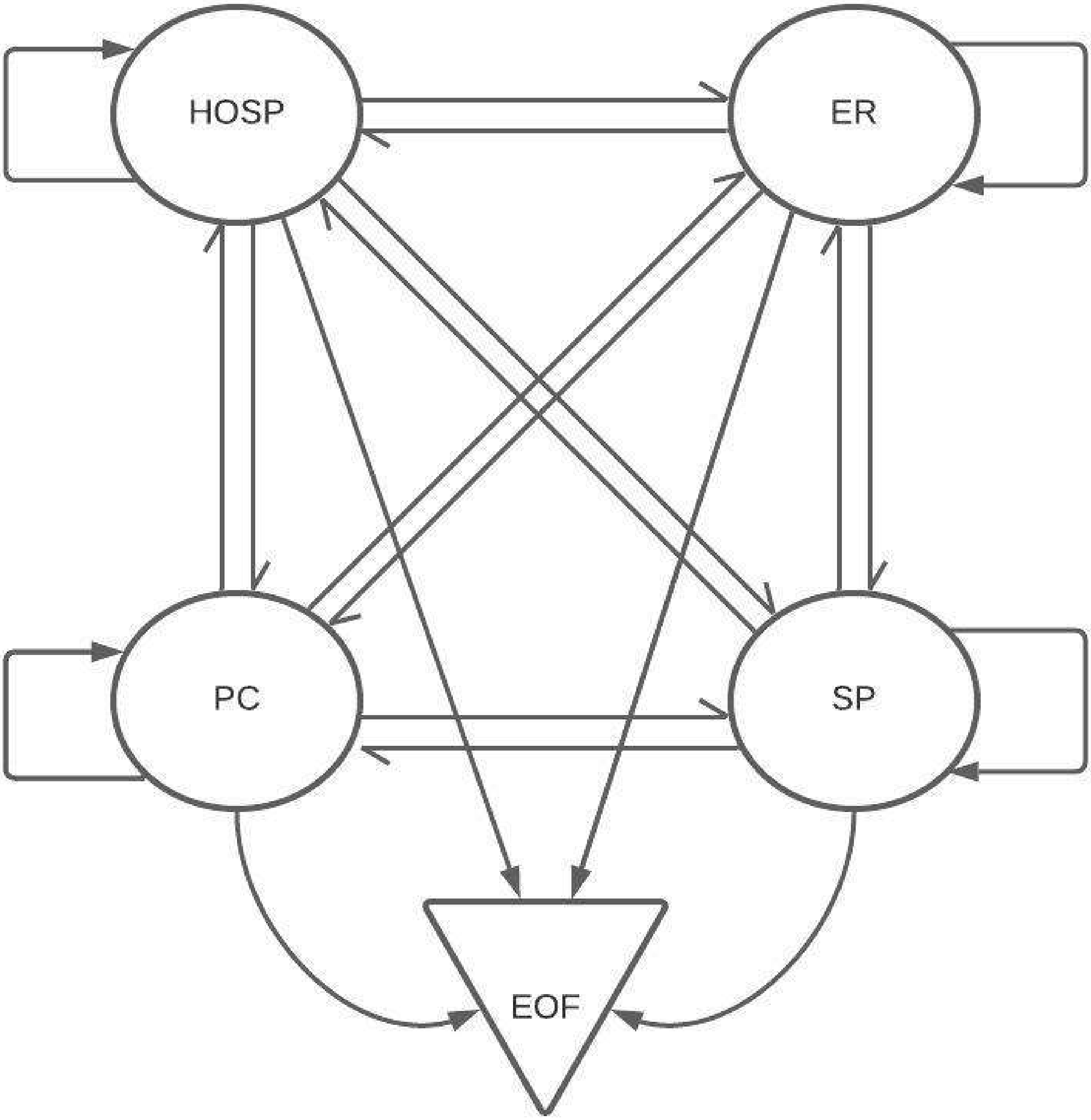

Supplementary Figure 3. Graphical representation of the Markov model for adults used in the study. The five states of the model represent each healthcare location where patients with AGE may receive medical assistance. The arrows represent the possible transitions between each of the Markov states up to a maximum of seven transitions, each associated with a certain probability. The last state (EOF) has no possibility of return. Hosp: Hospitalized patients; ER: Emergency Room, SP: Specialized Care; PC: Primary Care; EOF: End of Follow up.

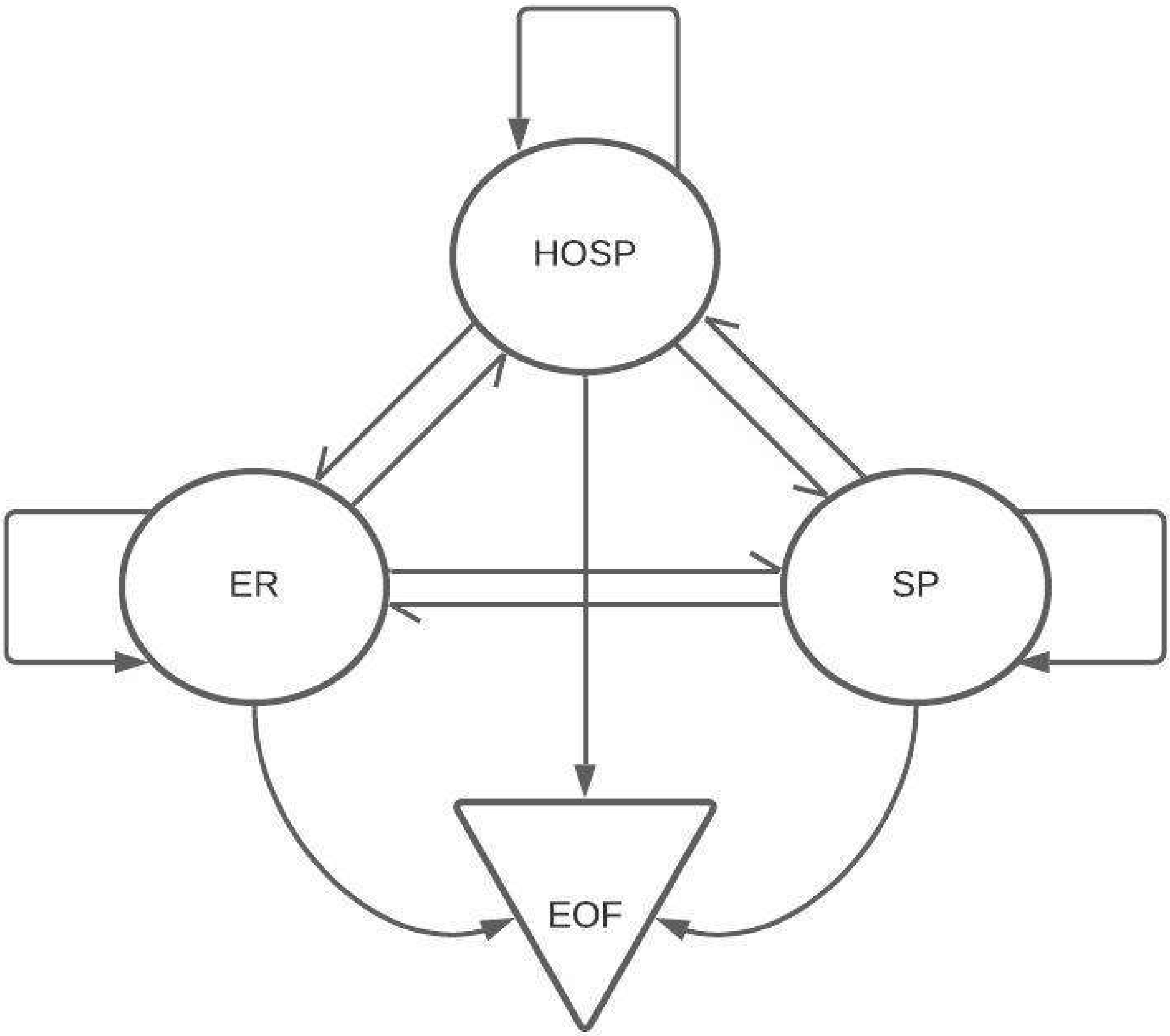

Supplementary Figure 4. Graphical representation of the Markov model for children used in the study. The four states of the model represents each healthcare location where patients with AGE may receive medical assistance. The arrows represent possible transitions between each Markov state up to a maximum of 7 transitions, each associated with a certain probability. The last state (EOF) has no possibility of return. Hosp: Hospitalized patients; ER: Emergency Room, SP: Specialized Care; EOF: End of Follow up.

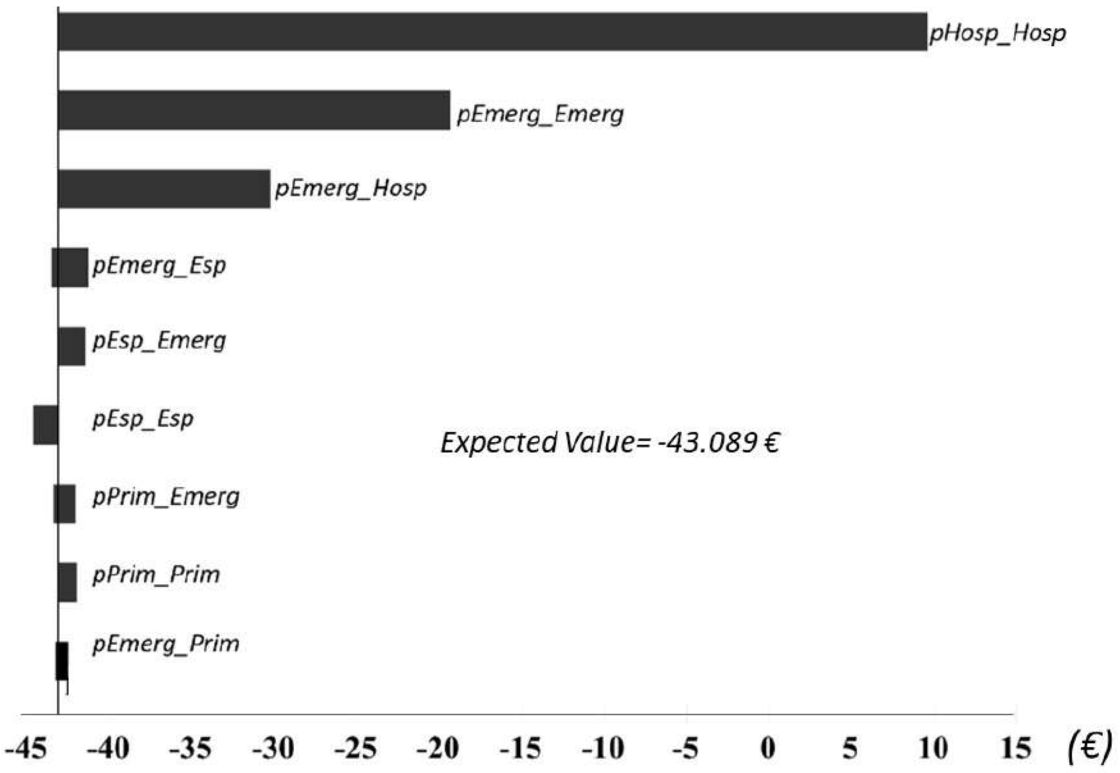

Supplementary Figure 5. Graphical representation of the tornado sensitivity analysis evaluating the relative weight of the variables included in the Markov model. The X-axis shows the profit in Euros of the molecular testing strategy (2018) compared to the non-molecular diagnostic approach (2017). Hosp: Hospitalized patients; Emerg: Emergency Room; Esp: Specialized Care; Prim: Primary Care.

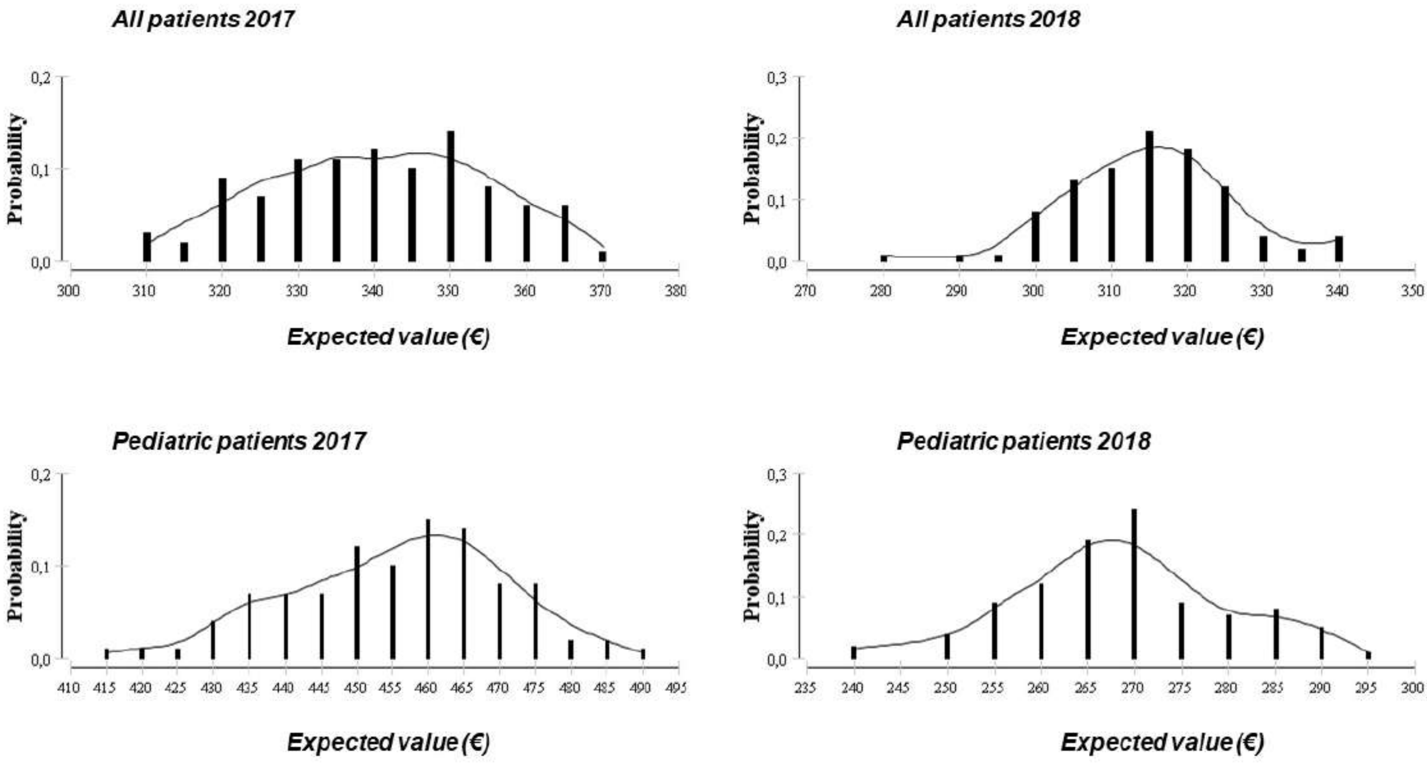

Supplementary Figure 6. Results from the Monte Carlo simulation, showing the distribution of expected costs considering all patients in 2017 (A) and 2018 (B), and pediatric patients in 2017 (C) and 2018 (D).
